# Assessment of Liver Stiffness in a Rat Model of Type 2 Diabetes Using Shear Wave Elastography

**DOI:** 10.3390/diagnostics16162518

**Published:** 2026-08-10

**Authors:** Fahad F. Al-mutairi, Mohammed H. Alhashmi, Aymn T. Abbas, Muhanad S. Hazazi, Almotazbillah A. Bedaiwi, Sara A. Alamoudi, Ali H. Almoris, Reham Y. Albaz, Wafaa S. Ramadan

**Affiliations:** 1 Department of Diagnostic Radiology, Faculty of Applied Medical Sciences, King Abdulaziz University, Jeddah 21589, Saudi Arabia; 2 Radiology Unit, King Fahd Medical Research Center, King Abdulaziz University, Jeddah 21589, Saudi Arabia; 3 Smart Medical Imaging Research Group, King Abdulaziz University, Jeddah 21589, Saudi Arabia; 4 Animal House Unit, King Fahd Medical Research Center, King Abdulaziz University, Jeddah 21589, Saudi Arabia; 5 Department of Medical Laboratory Sciences (MLS), Faculty of Applied Medical Sciences, King Abdulaziz University, Jeddah 21589, Saudi Arabia; 6 Special Infectious Agent Unit-BSL3, King Fahd Medical Research Center, King Abdulaziz University, Jeddah 21589, Saudi Arabia; 7 Pathology Department, King Abdulaziz University Hospital, Jeddah 21589, Saudi Arabia; 8 Central Laboratories, King Fahd Medical Research Center, King Abdulaziz University, Jeddah 22254, Saudi Arabia; 9 Department of Clinical Anatomy, Faculty of Medicine, King Abdulaziz University, Jeddah 21589, Saudi Arabia; 10 Stem Cell Research Unit, King Fahd Medical Research Center, King Abdulaziz University, Jeddah 21589, Saudi Arabia

**Keywords:** type 2 diabetes mellitus, shear wave elastography, liver stiffness, hepatic fibrosis, high-fat diet, streptozotocin, rat model, histopathology

## Abstract

**Background/Objectives:** Type 2 diabetes mellitus (T2DM) is associated with progressive hepatic alterations, including steatosis and fibrosis, which may affect liver mechanical properties. Shear wave elastography (SWE) is a non-invasive imaging technique that enables quantitative assessment of tissue stiffness; however, its application in diabetic animal models remains limited. This study aimed to evaluate liver elasticity using SWE in a high-fat diet (HFD) and streptozotocin (STZ)-induced rat model of T2DM, using histopathological examination as the reference standard. **Methods:** Twenty-four rats were randomly allocated to either a control group (n = 12) or a diabetic group (n = 12). Diabetes was induced using HFD feeding followed by low-dose STZ administration. Liver elasticity was assessed in vivo using SWE at two examination sessions. Shear wave velocity measurements were obtained from eight liver regions of interest and expressed in meters per second (m/s). Following euthanasia, liver tissues were subjected to histopathological evaluation using hematoxylin and eosin, Masson’s trichrome, and periodic acid–Schiff staining. **Results:** Diabetic rats demonstrated marked histopathological alterations, including macrovesicular and microvesicular steatosis, hepatocellular vacuolization, increased collagen deposition, and glycogen depletion. Collagen deposition was significantly greater in diabetic animals, while glycogen content was significantly reduced (3.70 ± 1.05 vs. 26.87 ± 2.87; *p* < 0.05). SWE revealed consistently higher liver shear wave velocities in diabetic rats compared with controls at both examination sessions. Although liver stiffness increased over time in both groups, these changes did not reach statistical significance. **Conclusions:** HFD/STZ-induced diabetes was associated with increased liver stiffness and histopathological evidence of hepatic injury and fibrosis. SWE may represent a feasible non-invasive approach for assessing diabetes-related changes in liver mechanical properties.

## 1. Introduction

Type 2 diabetes mellitus (T2DM) is one of the most prevalent metabolic disorders worldwide and is increasingly recognized as a major contributor to chronic liver disease [1]. Insulin resistance, hyperglycemia, and dysregulated lipid metabolism promote hepatic fat accumulation, inflammation, and fibrogenesis, leading to the development of metabolic dysfunction-associated steatotic liver disease (MASLD) [2,3]. The concurrence of T2DM and MASLD is associated with accelerated progression of liver fibrosis and increased risk of cirrhosis and liver-related complications [4,5]. Patients with type 2 diabetes mellitus are at increased risk of developing MASLD and progressive liver fibrosis, highlighting the importance of early diagnosis and disease monitoring, as recommended by the American Association for the Study of Liver Diseases (AASLD).

Liver fibrosis is characterized by excessive deposition of extracellular matrix components, particularly collagen, resulting in progressive architectural distortion and alterations in the mechanical properties of hepatic tissue [6]. Histopathological examination remains the reference standard for evaluating liver injury and fibrosis because it allows direct assessment of steatosis, inflammatory changes, collagen deposition, and glycogen content [7]. However, tissue sampling is invasive and unsuitable for repeated longitudinal assessment, creating a need for reliable non-invasive methods capable of monitoring disease progression over time. Although liver biopsy remains the reference standard for diagnosing hepatic fibrosis, it samples only a very small proportion of the liver parenchyma, making it susceptible to sampling variability and potential underestimation of disease severity. In contrast, elastography techniques such as SWE and magnetic resonance elastography (MRE) assess a substantially larger volume of liver tissue, thereby providing a more representative evaluation of tissue stiffness.

Ultrasound-based shear wave elastography (SWE) has emerged as a promising imaging technique for the quantitative assessment of tissue mechanical properties [8,9]. SWE measures the propagation velocity of mechanically induced shear waves within tissues, providing an indirect estimate of tissue stiffness. Increased liver stiffness has been associated with progressive fibrosis and extracellular matrix remodeling, making elastography an attractive non-invasive biomarker for liver disease assessment [10,11].

Experimental studies have demonstrated the feasibility of elastography for evaluating liver pathology in animal models. Lu et al. (2014) reported that real-time SWE successfully differentiated varying stages of liver fibrosis in rabbits and showed significant correlations with histological findings [12]. Similarly, Ogawa et al. (2016) demonstrated a strong relationship between liver stiffness measurements obtained using SWE and histopathological fibrosis severity in rats with metabolic dysfunction-associated steatohepatitis (MASH) confirming that elastographic measurements reflect underlying structural alterations within liver tissue [13]. These findings support the use of SWE as a non-invasive surrogate marker of histopathological changes.

Further evidence has been provided by studies investigating liver viscoelastic properties in experimental liver disease. Sugimoto et al. (2018) demonstrated the feasibility of measuring liver viscoelasticity in rat livers using shear wave ultrasonography and highlighted the ability of elastographic parameters to detect pathological tissue alterations [14]. Pi et al. (2021) subsequently showed that SWE-derived measurements were consistent with biomechanical properties obtained through dynamic mechanical analysis in a rat model of metabolic dysfunction-associated steatotic liver disease (MASLD), suggesting that elastography can accurately reflect changes in liver tissue mechanics [15]. More recently, Wu et al. (2023) reported that SWE measurements increased with the severity of hepatic steatosis in a rat model and demonstrated promising diagnostic performance for differentiating varying degrees of liver involvement [16].

The clinical relevance of liver stiffness assessment in diabetes has also been demonstrated in clinical studies. Sporea et al. (2016) reported increased liver stiffness values among patients with T2DM and ultrasound-confirmed hepatic steatosis, suggesting that elastography may provide valuable information regarding liver involvement in diabetic patients [17]. Despite these advances, the majority of experimental studies have focused on models of steatosis or metabolic dysfunction-associated steatotic liver disease (MASLD), while studies regarding liver elasticity changes in high-fat diet and streptozotocin (HFD/STZ)-induced T2DM models remain limited.

The HFD/STZ model is widely used because it reproduces key metabolic and histopathological features of T2DM, including insulin resistance, hyperglycemia, hepatic lipid accumulation, and fibrosis. Chronic metabolic disturbances stimulate hepatic stellate cell activation and collagen deposition, resulting in remodeling of the extracellular matrix and potentially altered tissue stiffness [5,18]. However, longitudinal assessment of these changes using SWE and their relationship with histopathological findings have not been fully elucidated.

Therefore, the present study aimed to evaluate liver elasticity using shear wave elastography at two sequential assessment sessions in a rat model of type 2 diabetes induced by a high-fat diet and streptozotocin. Histopathological examination was used as the reference standard to characterize hepatic steatosis, collagen deposition, and glycogen storage. We hypothesized that diabetic rats would demonstrate increased liver stiffness, reflected by higher shear wave velocities, corresponding to histopathological evidence of diabetic liver injury and fibrosis.

## 2. Materials and Methods

### 2.1. Animal Model and Experimental Design

This experimental study was conducted using 24 rats maintained under standardized laboratory conditions with controlled temperature, relative humidity (45–75%), and a 12 h light/dark cycle. Animals were provided ad libitum access to food and water and were acclimatized for one week before the commencement of the study. All experimental procedures were performed in accordance with the International Guidelines for the Care and Use of Laboratory Animals and were approved by the Unit of Biomedical Ethics, Faculty of Medicine, King Abdulaziz University (Approval No. 448-24, 30 December 2024).

Animals were randomly assigned into two groups (n = 12 per group): a control group and a diabetic group. The control group received a standard laboratory diet containing 5% fat, 52% carbohydrate, and 20% protein. The diabetic group was fed a high-fat diet consisting of 60% fat, 20% carbohydrate, and 20% protein (Research Diets Inc., New Brunswick, NJ, USA) to induce insulin resistance.

After four weeks of dietary intervention, diabetes was induced in the diabetic group by intraperitoneal injection of streptozotocin (STZ; Sigma-Aldrich, St. Louis, MO, USA) at a dose of 35 mg/kg body weight. STZ was freshly dissolved in 0.1 M sodium citrate buffer (pH 4.4) immediately prior to administration. One week following injection, fasting blood glucose concentrations were measured using a glucometer (Accu-Chek Performa, Roche Diagnostics, Indianapolis, IN, USA). Animals with fasting blood glucose levels below 200 mg/dL received a second STZ injection at the same dosage. Rats with fasting blood glucose concentrations ≥200 mg/dL were considered diabetic and included in the diabetic group.

Following confirmation of diabetes, animals remained on their respective diets for an additional four weeks before undergoing the first liver elasticity assessment (Session 1). The dietary intervention was subsequently continued for a further four weeks, after which a second liver elasticity assessment (Session 2) was performed. At the end of the experimental period, animals were anesthetized using inhaled isoflurane and euthanized. Liver tissues were immediately harvested for histopathological analysis.

### 2.2. Shear Wave Elastography

Liver elasticity was evaluated in vivo using ULTIMUS 9E ultrasound system (VINNO Technology Co., Ltd., Suzhou, China) equipped with shear wave elastography (SWE) technology. Prior to imaging, animals were anesthetized using inhaled isoflurane to minimize respiratory motion and movement-related artifacts. The abdominal region was shaved and depilated to improve acoustic coupling, and animals were positioned in dorsal recumbency on a heated platform to maintain body temperature during image acquisition.

Conventional B-mode ultrasonography was initially performed to identify the liver parenchyma and to avoid major vascular structures. Subsequently, SWE examinations were obtained through a right subcostal acoustic window. Imaging parameters were kept constant throughout all examinations to ensure consistency between animals and study sessions.

The ultrasound transducer was positioned parallel to the intercostal space to optimize shear wave propagation and measurement reliability. For each animal, eight independent regions of interest (ROIs) were acquired from homogeneous liver parenchyma while avoiding visible blood vessels and imaging artifacts. Shear wave velocity measurements were automatically generated by the ultrasound system and expressed in meters per second (m/s). The mean value of the eight measurements was calculated and used for statistical analysis.

All SWE examinations were performed by the same experienced operator, who was blinded to the experimental group allocation. To assess measurement repeatability between examination sessions, the coefficient of variation (CoV) was calculated according to the following formula: CoV (%) = (Standard Deviation/Mean) × 100. Lower CoV values were considered indicative of greater measurement consistency between repeated acquisitions.

### 2.3. Histopathological Evaluation

Following euthanasia, liver specimens were immediately excised and fixed in 4% paraformaldehyde. Fixed tissues were processed routinely, embedded in paraffin, and sectioned at a thickness of 4 μm. Histological evaluation was performed using hematoxylin and eosin (H&E), Masson’s trichrome, and periodic acid–Schiff (PAS) staining techniques.

Hematoxylin and eosin staining was used to assess the general hepatic architecture and to identify structural alterations, including hepatocellular vacuolization and steatosis. Masson’s trichrome staining was performed to evaluate collagen deposition and hepatic fibrotic changes. Periodic acid–Schiff staining was used to assess glycogen distribution and storage within hepatocytes. Histological sections were examined by an experienced pathologist who was blinded to the experimental groups. Representative photomicrographs were captured using an Olympus BX53 microscope equipped with a DP73 digital camera system (Olympus Corporation, Tokyo, Japan).

### 2.4. Statistical Analysis

No formal a priori power calculation was performed because this was an exploratory experimental animal study. The sample size of 24 rats (12 per group) was selected based on previous shear wave elastography studies using comparable rodent models, while considering ethical principles aimed at minimizing animal use and ensuring sufficient statistical power to detect biologically meaningful differences between groups. Statistical analyses were performed using IBM SPSS Statistics version 22.0 (IBM Corp., Armonk, NY, USA). Continuous variables are presented as mean ± standard deviation (SD). Comparisons between diabetic and control groups were performed using the independent-samples *t*-test. Differences in liver elasticity measurements between the two examination sessions were also evaluated. Statistical significance was defined as a two-tailed *p*-value < 0.05.

## 3. Results

### 3.1. Histopathological Findings

**Figure 1:**
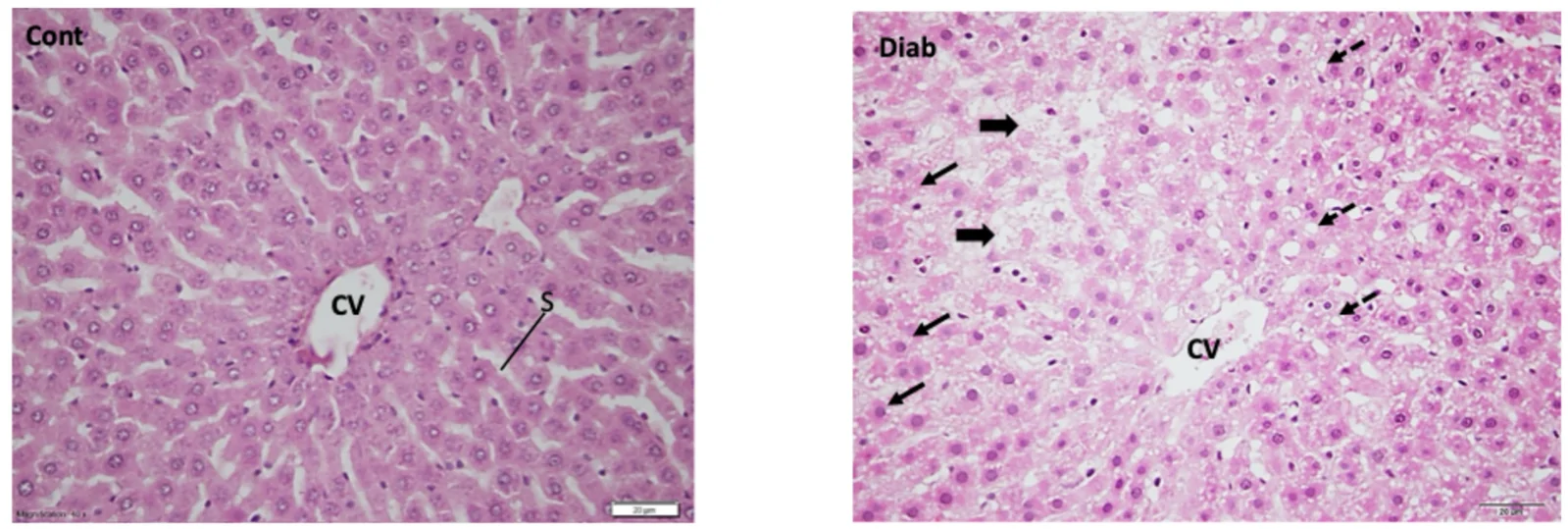
Photomicrographs of sections of livers of the Cont group showing well-organised liver architecture with hepatocytes separated by sinusoids (S) radiating from the central vein (CV). Diab section showing distorted structure with hepatocytes: microvesicles (arrow), macrovesicles (dashed arrow), and intense vacuolization of hepatocytes (thick arrow). (H&E ×400).

Histological examination of liver sections stained with hematoxylin and eosin (H&E) demonstrated normal hepatic architecture in the control group. Hepatocytes were arranged in well-organized hepatic cords radiating from the central vein and separated by regular hepatic sinusoids (Figure 1).

In contrast, liver sections obtained from diabetic rats exhibited marked histopathological alterations, including distortion of the normal hepatic architecture, extensive hepatocellular vacuolization, and both microvesicular and macrovesicular fatty infiltration. These changes were absent in the control group and were consistent with diabetes-associated hepatic injury (Figure 1).

### 3.2. Assessment of Hepatic Fibrosis

**Figure 2:**
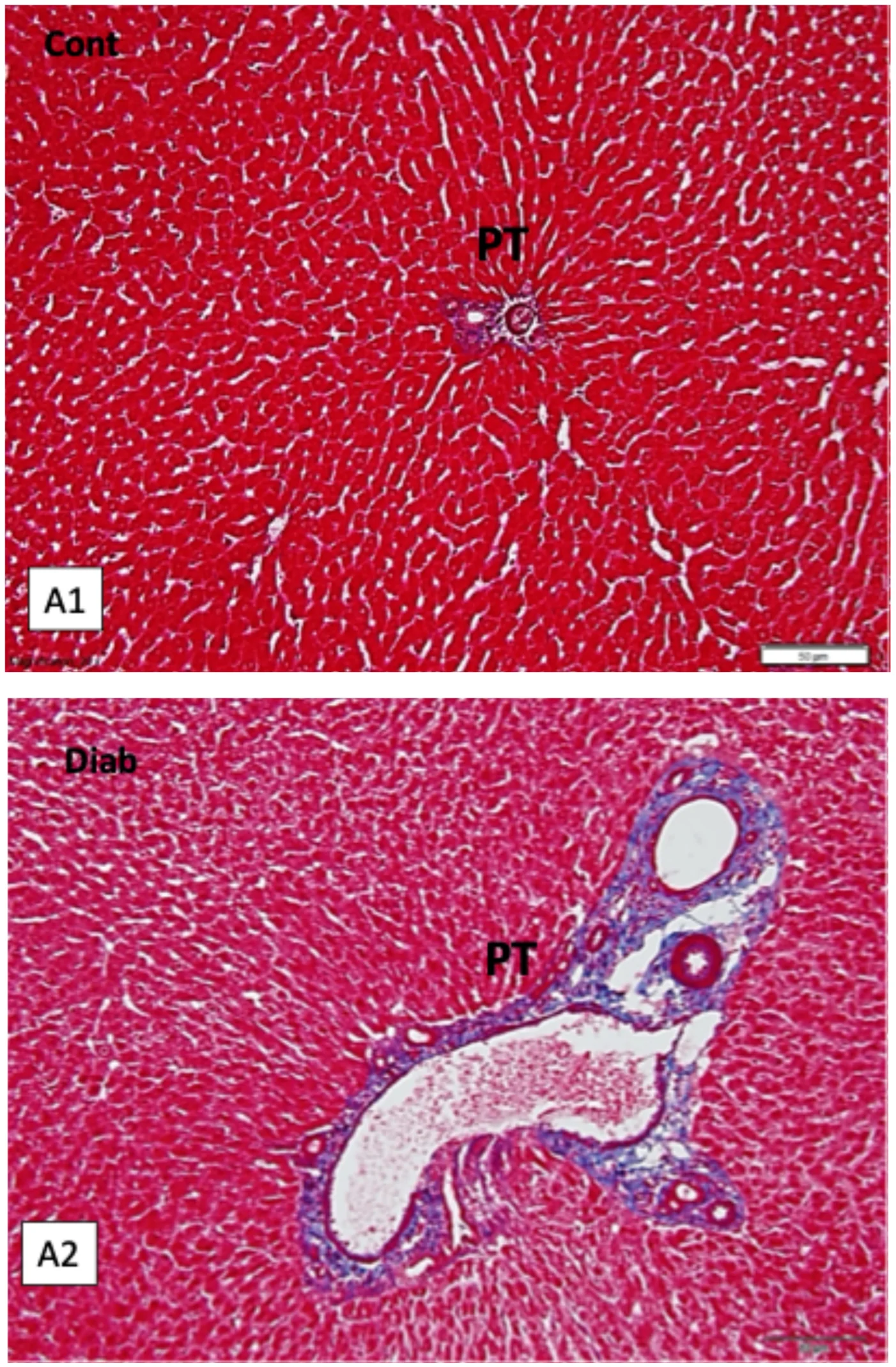

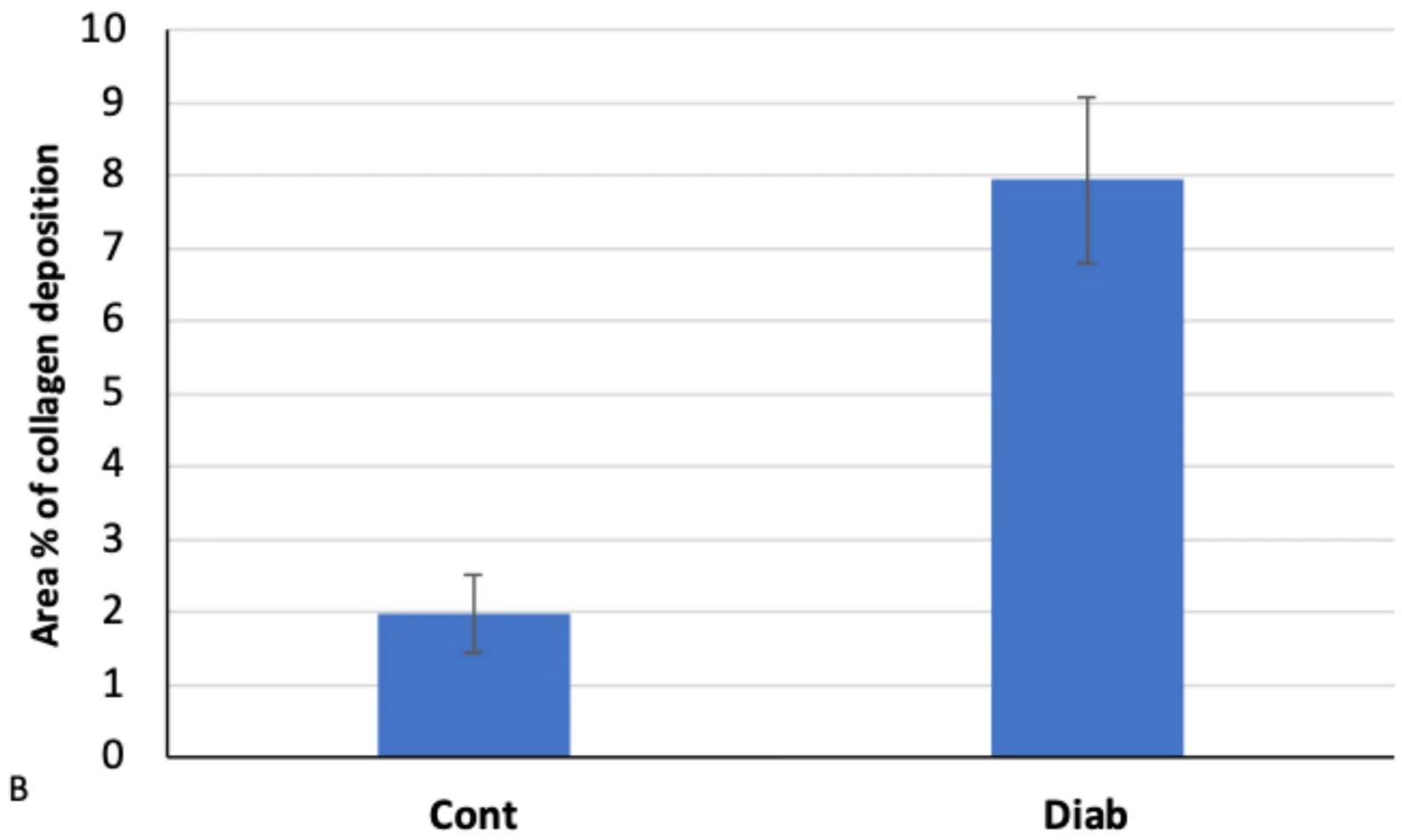
Photomicrographs of Masson’s trichrome-stained liver sections showing minimal collagen deposition in the control group (Cont; (**A1**)), whereas the diabetes group (Diab; (**A2**)) exhibited a marked increase in collagen deposition in the periportal regions. Collagen fibers are stained blue. (**B**) Quantitative analysis demonstrating a significant increase in the percentage area of collagen deposition in the Diab group consistent with the increased periportal collagen accumulation observed histologically. Data are presented as mean ± SD. An independent-samples *t*-test was used for comparison between the two groups. *p* < 0.05 was considered statistically significant. PT: portal tract; Cont: control; Diab: diabetes. Masson’s trichrome staining, ×200.

Masson’s trichrome staining revealed minimal collagen deposition within liver sections from the control group. In contrast, liver sections from diabetic rats demonstrated prominent periportal collagen accumulation (Figure 2(A1,A2)). Quantitative analysis showed a significant increase in collagen area percentage in the diabetic group compared with the control group (*p* < 0.05) (Figure 2B), indicating increased hepatic fibrotic remodeling in diabetic animals.

### 3.3. Assessment of Hepatic Glycogen Storage

**Figure 3:**
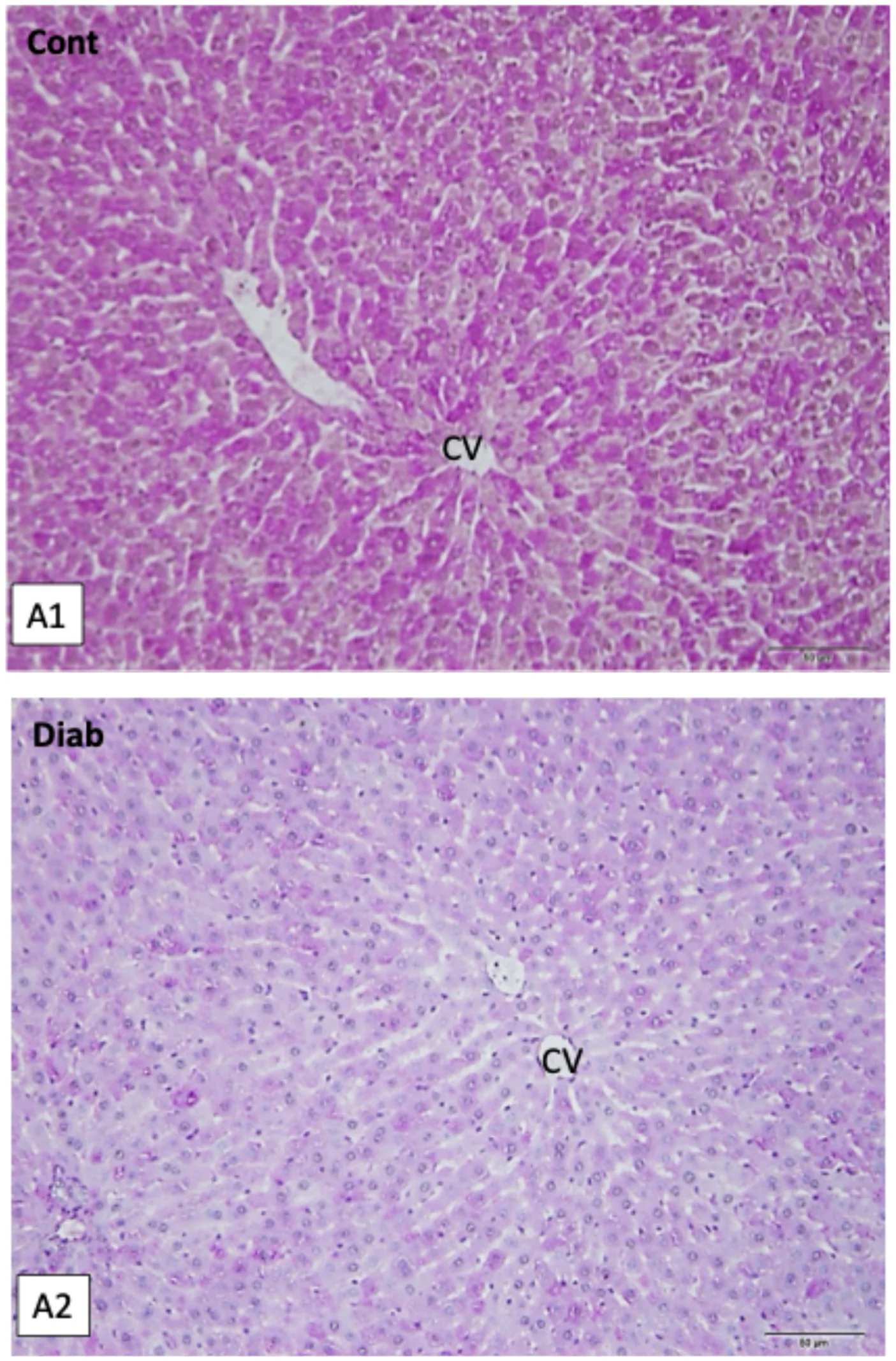

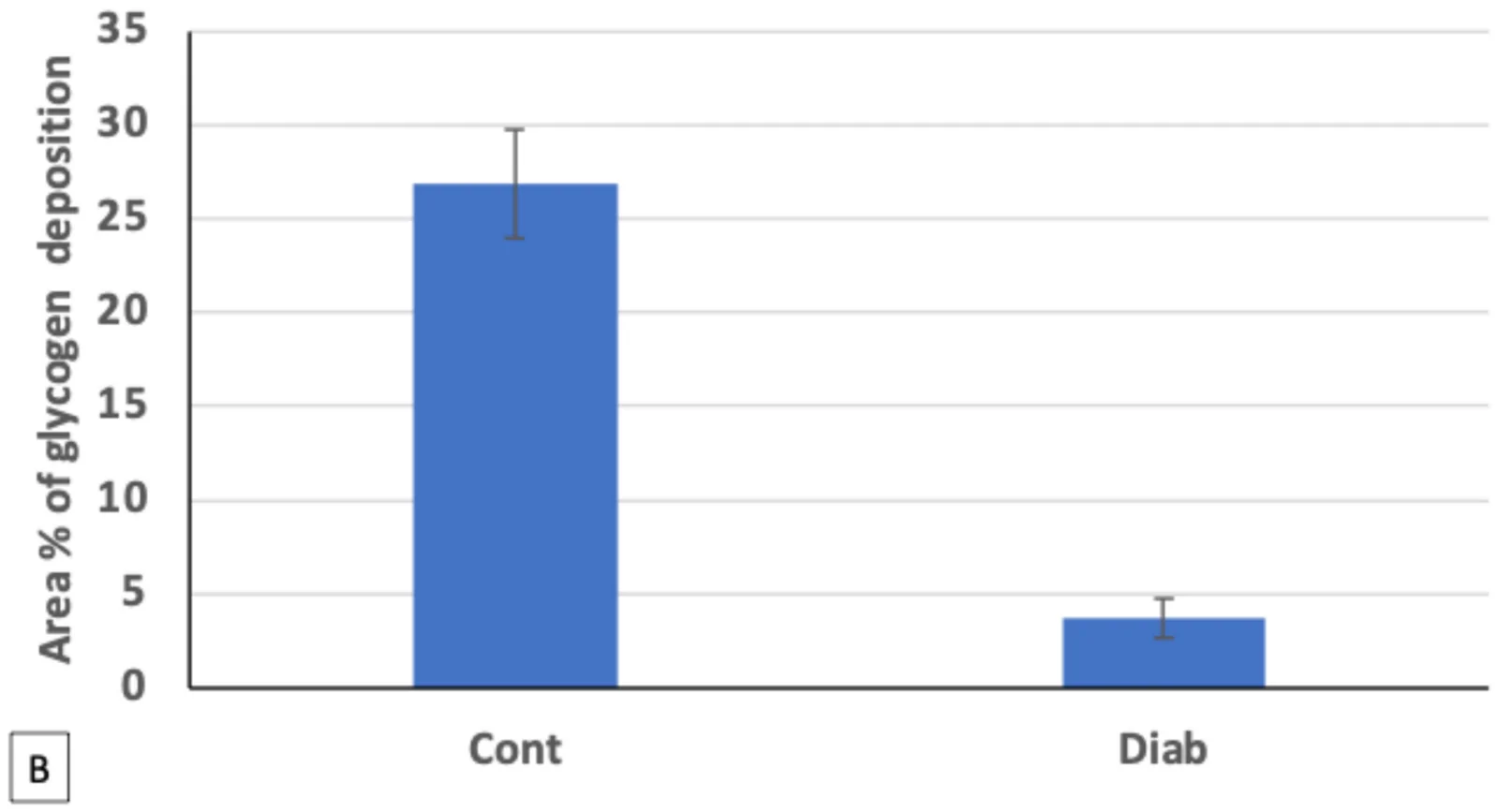
Photomicrographs of PAS-stained liver sections showing significant PAS-stained glycogen in hepatocytes of the control group (Cont; (**A1**)), whereas the diabetes group (Diab; (**A2**)) revealed marked depletion in the pericentral regions. Glycogen is stained deep purple. (**B**) Quantitative analysis demonstrating a significant decrease in the percentage area of glycogen deposition in the (Diab) group consistent with the pericentral depletion observed histologically. Data are presented as mean ± SD. An independent-samples *t*-test was used for comparison between the two groups. *p* < 0.05 was considered statistically significant. PT: portal tract; Cont: control; Diab: diabetes. Masson’s trichrome staining, ×200.

Periodic acid–Schiff (PAS) staining demonstrated abundant glycogen deposition within hepatocytes of the control group. Conversely, liver sections from diabetic rats showed marked depletion of PAS-positive glycogen staining (Figure 3(A1,A2)). Quantitative analysis revealed a significant reduction in glycogen content in the diabetic group compared with controls. The mean glycogen area percentage was 3.70 ± 1.05 in diabetic rats compared with 26.87 ± 2.87 in the control group (*p* < 0.05) (Figure 3B).

### 3.4. Shear Wave Elastography Findings

**Figure 4:**
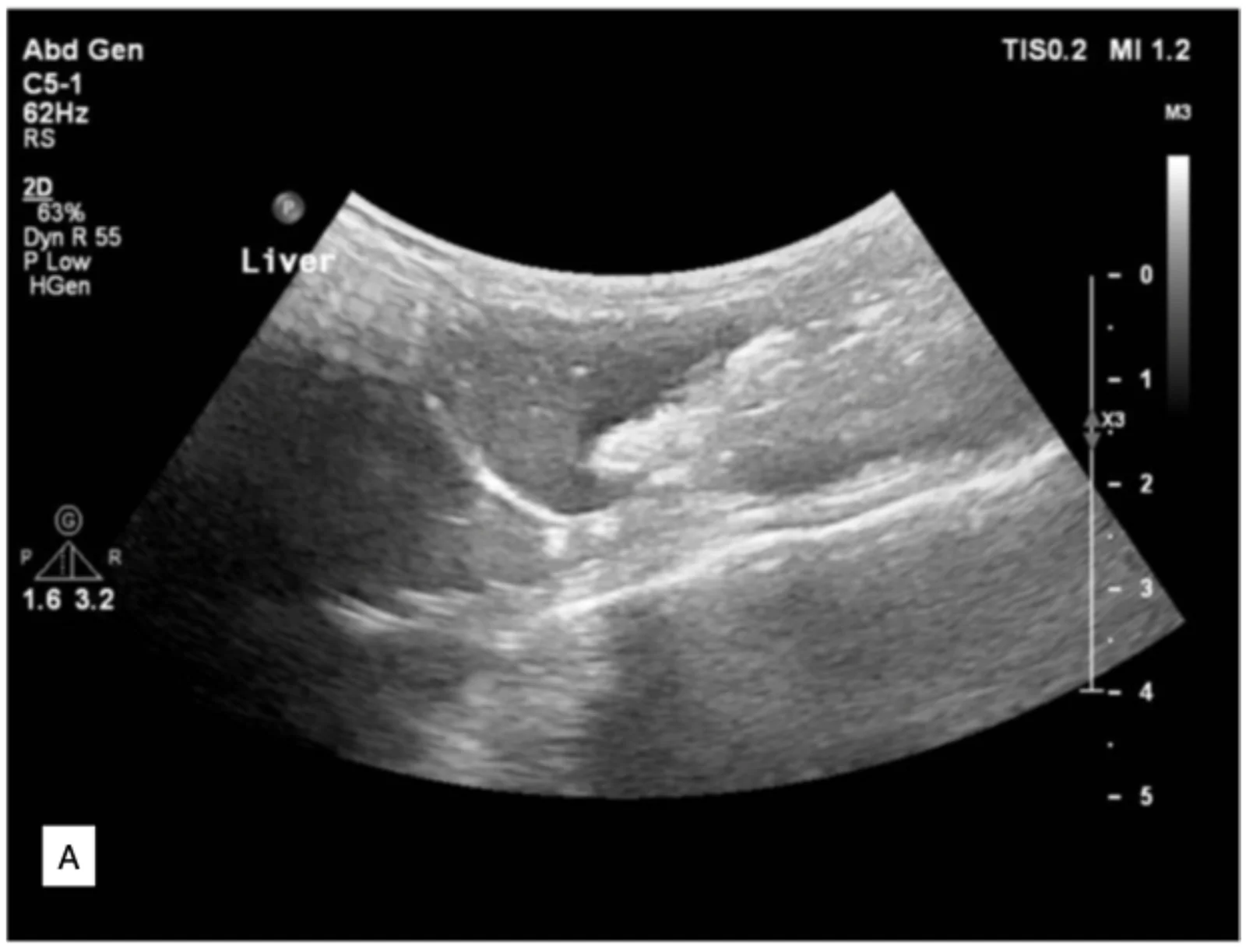

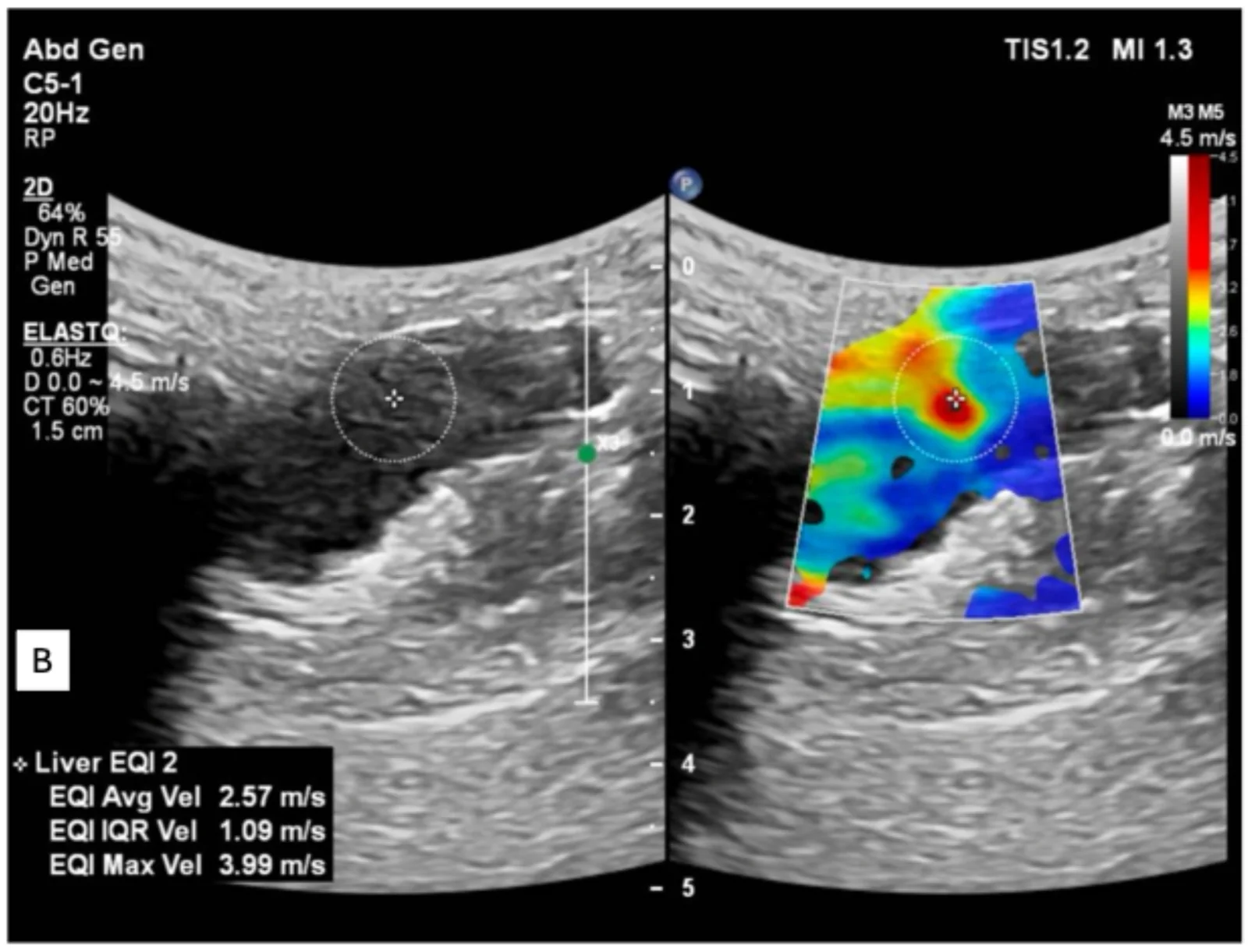

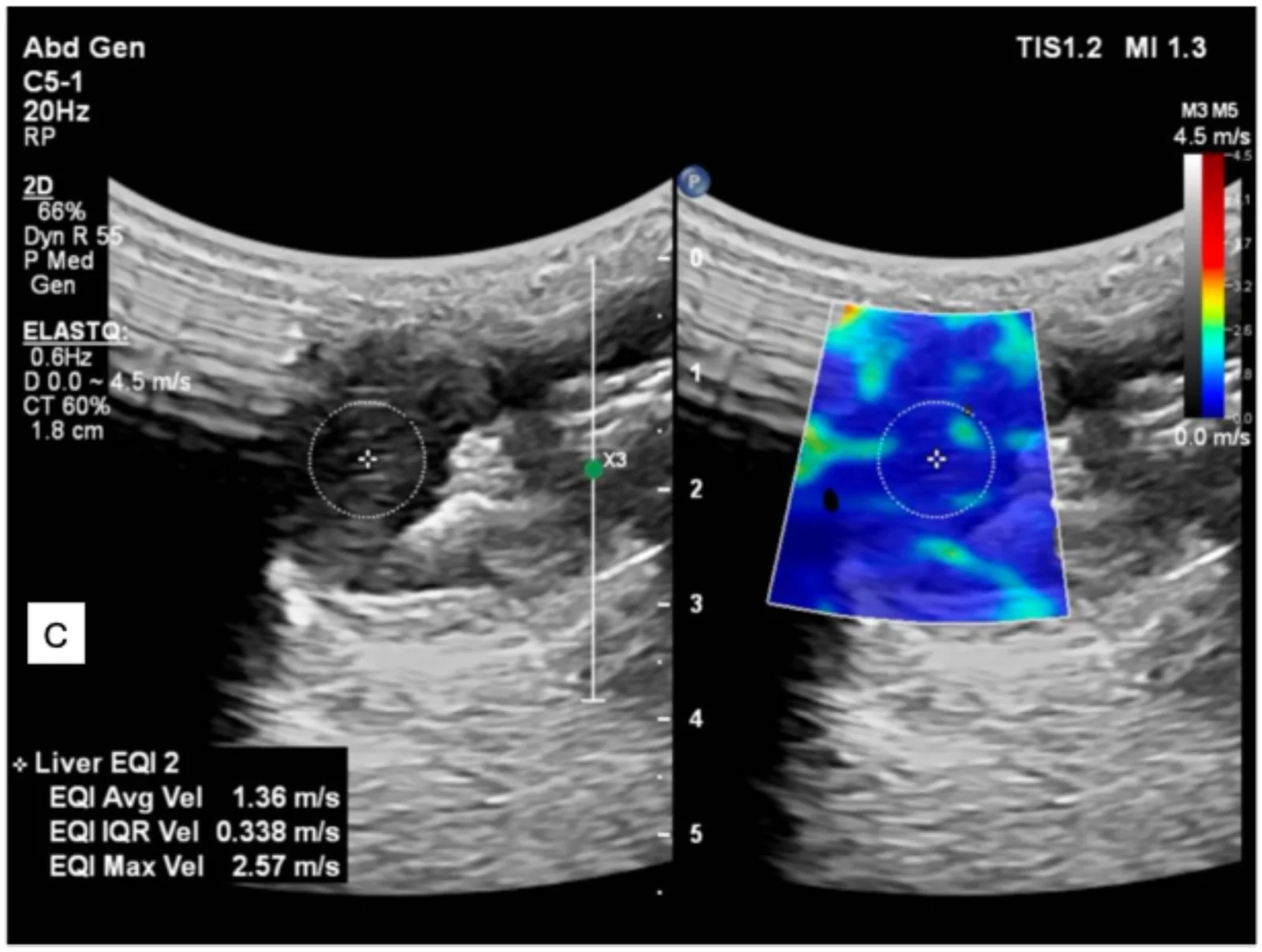
Representative shear wave elastography images obtained from B-mode ultrasound (**A**), diabetic rat (**B**) and control rat (**C**). The colour elastogram demonstrates the distribution of tissue stiffness within the liver parenchyma. Circular regions of interest (ROIs) were positioned within homogeneous liver tissue while avoiding major blood vessels and imaging artefacts. The diabetic liver demonstrates higher shear wave velocity values, consistent with increased liver stiffness.

**Figure 5:**
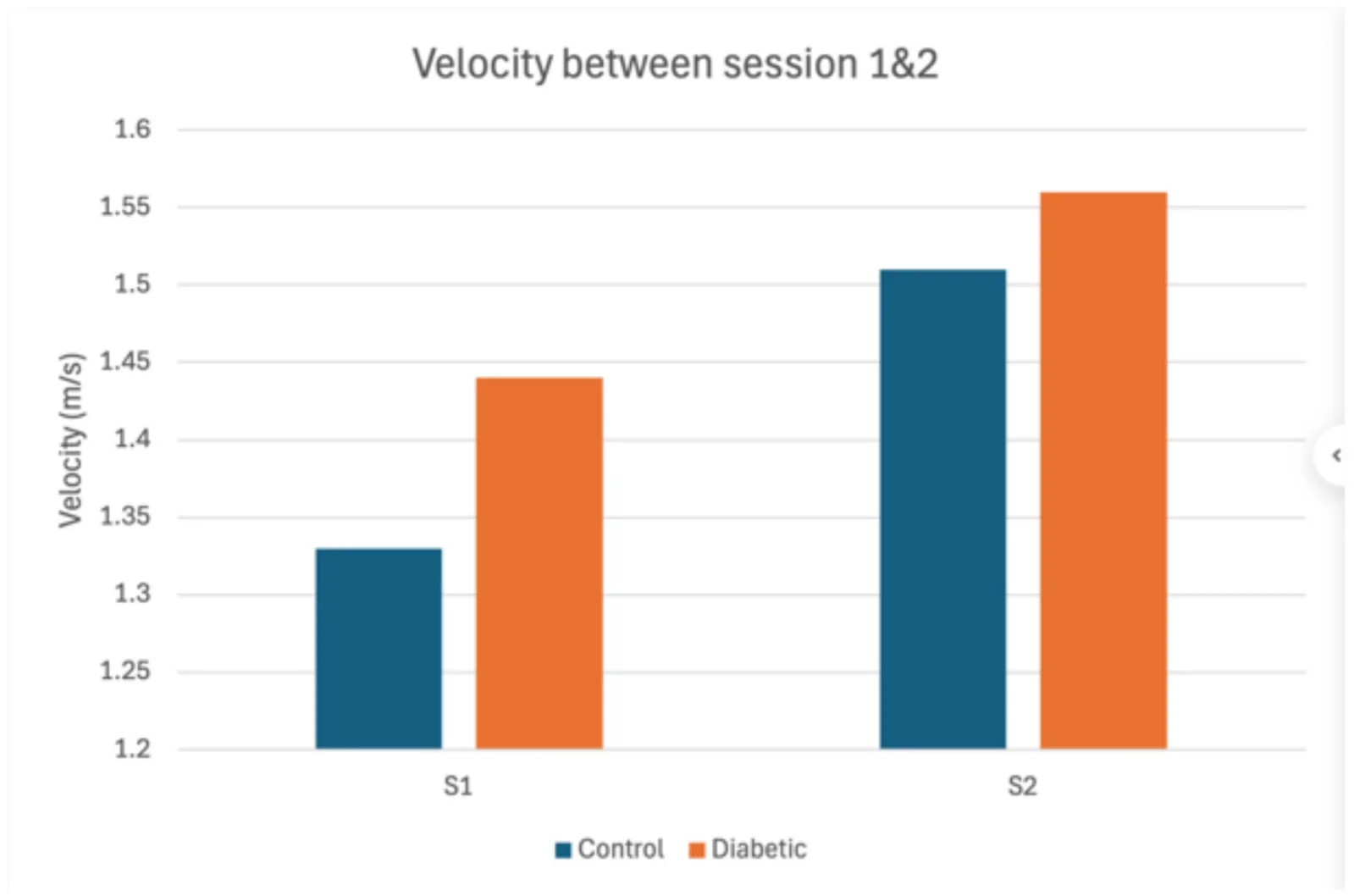
The average velocities of liver tissue in both groups in Session 2 were higher than those in Session 1, but with no statistical difference: S1 (1.33 vs. 1.51; *p* = 0.41) and S2 (1.51 vs. 1.56; *p* = 0.73). It can clearly be seen that the liver tissue in the Diabetic group in both sessions exhibits higher velocities than the Control group.

The animals were not fasted prior to the shear wave elastography examinations. Liver elasticity was assessed using shear wave elastography at two separate examination sessions. Representative SWE measurements are illustrated in Figure 4. At both examination sessions, diabetic rats exhibited higher liver shear wave velocities compared with control animals, indicating increased liver stiffness in the diabetic group (Figure 5). Assessment of measurement repeatability demonstrated acceptable consistency of SWE measurements in both groups. In Session 1, the coefficient of variation was 22% in the control group and 19% in the diabetic group. Repeatability improved during Session 2, with CoV values of 17% and 15% in the control and diabetic groups, respectively, indicating greater measurement stability during the second examination.

### 3.5. Longitudinal Assessment of Liver Elasticity

The mean liver shear wave velocity increased between the two examination sessions in both groups. In the control group, mean velocity increased from 1.33 m/s during Session 1 to 1.51 m/s during Session 2. Similarly, in the diabetic group, mean velocity increased from 1.51 m/s to 1.56 m/s over the same period. However, these within-group increases did not reach statistical significance (Control: *p* = 0.41; Diabetic: *p* = 0.73). Despite the absence of significant temporal changes, liver shear wave velocities remained consistently higher in diabetic rats than in control animals at both examination sessions (Figure 5). Overall, the elastographic findings were accompanied by histopathological evidence of hepatic steatosis, increased collagen deposition, and depletion of glycogen stores in diabetic animals.

## 4. Discussion

The present study investigated liver elasticity using shear wave elastography in a high-fat diet and streptozotocin-induced rat model of type 2 diabetes mellitus, with histopathological examination serving as the reference standard. The principal findings were that diabetic rats exhibited higher liver shear wave velocities than control animals at both examination sessions and demonstrated histopathological evidence of hepatic injury characterized by steatosis, increased collagen deposition, and depletion of glycogen stores. Collectively, these findings suggest that diabetes-induced structural alterations within the liver are accompanied by measurable changes in tissue mechanical properties.

Histological examination revealed marked hepatic abnormalities in diabetic animals, including macrovesicular and microvesicular steatosis, hepatocellular vacuolization, and disruption of normal liver architecture. These observations are consistent with previous reports demonstrating that insulin resistance and chronic hyperglycemia promote hepatic lipid accumulation through increased lipolysis, enhanced free fatty acid delivery to the liver, and impaired fatty acid oxidation [4,19,20]. The resulting metabolic disturbances contribute to progressive hepatic injury and remodeling. A notable finding of the present study was the significant increase in collagen deposition observed in diabetic animals compared with controls. Hepatic fibrosis is a recognized consequence of chronic metabolic dysfunction and is mediated by activation of hepatic stellate cells and excessive extracellular matrix production [18,21]. Previous clinical studies have shown that individuals with type 2 diabetes are at increased risk of fibrosis progression and advanced liver disease [5,22,23]. The increased collagen accumulation observed in our diabetic group is therefore consistent with the known pathological effects of diabetes on hepatic tissue.

Although magnetic resonance elastography (MRE) generally demonstrates superior diagnostic performance for detecting early-stage fibrosis, particularly when distinguishing minimal fibrosis from normal liver tissue, SWE remains an attractive alternative because of its lower cost, greater accessibility, shorter examination time, and suitability for repeated longitudinal assessments in both experimental and clinical settings.

The SWE findings demonstrated higher liver shear wave velocities in diabetic animals at both examination sessions, indicating increased tissue stiffness. These findings are biologically plausible given the histopathological evidence of fibrosis observed in the diabetic group. Previous studies have demonstrated that liver stiffness is closely related to extracellular matrix accumulation and fibrosis severity [10,24,25]. Our findings therefore support the concept that elastographic measurements may reflect underlying structural changes occurring within diabetic liver tissue.

The present findings are in agreement with previous experimental studies investigating elastography in liver disease models. Lu et al. (2014) reported that shear wave elastography could differentiate varying stages of fibrosis and showed significant associations with histological findings [12]. Similarly, Ogawa et al. (2016) demonstrated a strong relationship between liver stiffness measurements and histopathological fibrosis severity in rats with metabolic dysfunction-associated steatohepatitis (MASH) [13]. Sugimoto et al. (2018) further demonstrated the feasibility of assessing liver viscoelastic properties using shear wave ultrasonography in rat models, while Pi et al. (2021) reported that elastographic measurements reflected biomechanical alterations identified through dynamic mechanical analysis [14,15]. More recently, Wu et al. (2023) demonstrated that SWE measurements increased with the severity of hepatic steatosis in experimental liver disease [16]. Collectively, these studies support the ability of SWE to detect pathological alterations in liver tissue and provide a framework for interpreting the findings of the present study.

The observed reduction in hepatic glycogen content in diabetic rats is also consistent with the metabolic consequences of insulin deficiency and insulin resistance. Impaired glycogen synthesis and storage have been previously reported in diabetic liver tissue and may contribute to altered hepatic metabolism and structural integrity [26,27]. Although the relationship between glycogen depletion and tissue stiffness remains incompletely understood, these findings further confirm the presence of significant metabolic and histological alterations in the diabetic liver.

Interestingly, although liver shear wave velocities increased between the two examination sessions in both groups, these changes did not reach statistical significance. This finding may indicate that the duration between assessments was insufficient to detect substantial progression in tissue stiffness or may reflect the relatively small sample size of the study. Nevertheless, the consistently higher shear wave velocities observed in diabetic animals across both sessions suggest persistent alterations in liver mechanical properties associated with diabetes-induced hepatic injury.

The observed CoV values ranged from 15% to 22%, demonstrating acceptable repeatability of SWE measurements in this experimental setting. Furthermore, the lower CoV values observed during the second examination session suggest improved measurement consistency, possibly reflecting increased operator familiarity and stabilization of the imaging protocol.

The present findings support the potential role of SWE as a non-invasive imaging biomarker for monitoring diabetes-associated liver disease. As liver stiffness reflects progressive structural alterations associated with steatosis and fibrosis, SWE may facilitate longitudinal assessment of disease progression without repeated invasive biopsy. These experimental observations provide a foundation for future translational studies investigating the clinical utility of SWE in patients with MASLD and diabetes.

The present study has several limitations. First, this was an experimental animal study with a relatively small sample size. Second, serial histopathological assessment was not performed because repeated liver biopsy is not feasible in the same animals. Furthermore, although the HFD/STZ rat model reproduces several important metabolic and histopathological characteristics of MASLD associated with type 2 diabetes, animal models cannot fully replicate the complexity of human disease. In addition, although SWE examinations were performed using a standardized imaging protocol by an experienced operator, the technique remains partially operator-dependent. Finally, validation in additional experimental models and prospective clinical studies is required before these findings can be translated into routine clinical practice. Despite these limitations, the study demonstrates the feasibility of using shear wave elastography to assess liver mechanical properties in a high-fat diet and streptozotocin-induced model of type 2 diabetes. The findings indicate that increased liver stiffness is accompanied by histopathological evidence of steatosis and fibrosis, supporting the potential value of SWE as a non-invasive tool for monitoring diabetes-related liver alterations in experimental settings.

## 5. Conclusions

This study demonstrates that SWE can detect diabetes-associated increases in liver stiffness in an HFD/STZ rat model, with findings supported by histopathological analysis. These results highlight the translational potential of SWE as a non-invasive biomarker for monitoring liver disease progression. Future studies should include larger longitudinal cohorts, integration with additional serum and tissue biomarkers, and validation across different experimental models and clinical populations.

## Data Availability

The raw data supporting the conclusions of this article will be made available by the authors on request.

## References

[1] Qi X., Li J., Caussy C., Teng G.J., Loomba R. (2026). Epidemiology, screening, and co-management of type 2 diabetes mellitus and metabolic dysfunction–associated steatotic liver disease. Hepatology.

[2] Truong X.T., Lee D.H. (2025). Hepatic insulin resistance and steatosis in metabolic dysfunction-associated steatotic liver disease: New insights into mechanisms and clinical implications. Diabetes Metab. J..

[3] Sandireddy R., Sakthivel S., Gupta P., Behari J., Tripathi M., Singh B.K. (2024). Systemic impacts of metabolic dysfunction-associated steatotic liver disease (MASLD) and metabolic dysfunction-associated steatohepatitis (MASH) on heart, muscle, and kidney related diseases. Front. Cell Dev. Biol..

[4] Bugianesi E., Gastaldelli A., Vanni E., Gambino R., Cassader M., Baldi S., Ponti V., Pagano G., Ferrannini E., Rizzetto M. (2005). Insulin resistance in non-diabetic patients with non-alcoholic fatty liver disease: Sites and mechanisms. Diabetologia.

[5] Huang D.Q., Wilson L.A., Behling C., Kleiner D.E., Kowdley K.V., Dasarathy S., Amangurbanova M., Terrault N.A., Diehl A.M., Chalasani N. (2023). Fibrosis progression rate in biopsy-proven nonalcoholic fatty liver disease among people with diabetes versus people without diabetes: A multicenter study. Gastroenterology.

[6] Kisseleva T., Brenner D. (2021). Molecular and cellular mechanisms of liver fibrosis and its regression. Nat. Rev. Gastroenterol. Hepatol..

[7] Brunt E.M., Tiniakos D.G. (2010). Histopathology of nonalcoholic fatty liver disease. World J. Gastroenterol. WJG.

[8] Almutairi F., Alyami J. (2024). Feasibility of point shear wave elastography for evaluating renal cortical thickness: A prospective study. Curr. Med. Imaging.

[9] Almutairi F.F. (2024). The feasibility of point shear wave elastography (pSWE) in the assessment of pancreas stiffness in diabetic patients and healthy volunteers. PLoS ONE.

[10] Yeh W.-C., Li P.-C., Jeng Y.-M., Hsu H.-C., Kuo P.-L., Li M.-L., Yang P.-M., Lee P.H. (2002). Elastic modulus measurements of human liver and correlation with pathology. Ultrasound Med. Biol..

[11] Fang C., Sidhu P.S. (2020). Ultrasound-based liver elastography: Current results and future perspectives. Abdom. Radiol..

[12] Lu Y., Wei J., Tang Y., Yuan Y., Huang Y., Zhang Y., Li Y. (2014). Evaluation of fatty liver fibrosis in rabbits using real-time shear wave elastography. Exp. Ther. Med..

[13] Ogawa S., Moriyasu F., Yoshida K., Oshiro H., Kojima M., Sano T., Furuichi Y., Kobayashi Y., Nakamur I., Sugimoto K. (2016). Relationship between liver tissue stiffness and histopathological findings analyzed by shear wave elastography and compression testing in rats with non-alcoholic steatohepatitis. J. Med. Ultrason..

[14] Sugimoto K., Moriyasu F., Oshiro H., Takeuchi H., Yoshimasu Y., Kasai Y., Furuichi Y., Itoi T. (2018). Viscoelasticity measurement in rat livers using shear-wave US elastography. Ultrasound Med. Biol..

[15] Pi Z., Wang M., Lin H., Guo Y., Chen S., Diao X., Xia H., Liu G., Zeng J., Zhang X. (2021). Viscoelasticity measured by shear wave elastography in a rat model of nonalcoholic fatty liver disease: Comparison with dynamic mechanical analysis. BioMed Eng. OnLine.

[16] Wu Y., Liu Q., Wang Y., Li F., Chan L.W.C., Wen Y., Yang F., Xiang Q., Luo P., Lei P. (2023). Diagnostic efficiency on ultrasound shear wave elastography in evaluation of steatosis severity for non-alcoholic fatty liver disease: A rat model. Eur. J. Med. Res..

[17] Sporea I., Mare R., Lupușoru R., Sima A., Șirli R., Popescu A., Timar R. (2016). Liver Stiffness Evaluation by Transient Elastography in Type 2 Diabetes Mellitus Patients with Ultrasound-proven Steatosis. J. Gastrointest. Liver Dis..

[18] Kristensen D.B., Kawada N., Imamura K., Miyamoto Y., Tateno C., Seki S., Kuroki T., Yoshizato K. (2000). Proteome analysis of rat hepatic stellate cells. Hepatology.

[19] Cao X., Wang N., Yang M., Zhang C. (2025). Lipid accumulation and insulin resistance: Bridging metabolic dysfunction-associated fatty liver disease and chronic kidney disease. Int. J. Mol. Sci..

[20] Perry R.J., Samuel V.T., Petersen K.F., Shulman G.I. (2014). The role of hepatic lipids in hepatic insulin resistance and type 2 diabetes. Nature.

[21] Horn P., Tacke F. (2024). Metabolic reprogramming in liver fibrosis. Cell Metab..

[22] Lomonaco R., Godinez Leiva E., Bril F., Shrestha S., Mansour L., Budd J., Romero J.P., Schmidt S., Chang K.-L., Samraj G. (2021). Advanced liver fibrosis is common in patients with type 2 diabetes followed in the outpatient setting: The need for systematic screening. Diabetes Care.

[23] Ciardullo S., Monti T., Perseghin G. (2021). High prevalence of advanced liver fibrosis assessed by transient elastography among US adults with type 2 diabetes. Diabetes Care.

[24] Ortiz C., Schierwagen R., Schaefer L., Klein S., Trepat X., Trebicka J. (2021). Extracellular matrix remodeling in chronic liver disease. Curr. Tissue Microenviron. Rep..

[25] McQuitty C.E., Williams R., Chokshi S., Urbani L. (2020). Immunomodulatory role of the extracellular matrix within the liver disease microenvironment. Front. Immunol..

[26] Burrage L.C., Madan S., Li X., Ali S., Mohammad M., Stroup B.M., Jiang M.-M., Cela R., Bertin T., Jin Z. (2020). Chronic liver disease and impaired hepatic glycogen metabolism in argininosuccinate lyase deficiency. JCI Insight.

[27] Jiang S., Young J.L., Wang K., Qian Y., Cai L. (2020). Diabetic-induced alterations in hepatic glucose and lipid metabolism: The role of type 1 and type 2 diabetes mellitus. Mol. Med. Rep..

